# Age-related differences in the goals and concerns that motivate real-life prospective memory tasks

**DOI:** 10.1371/journal.pone.0216888

**Published:** 2019-06-03

**Authors:** Suzanna L. Penningroth, Walter D. Scott

**Affiliations:** Psychology Department, Washington State University, Pullman, WA, United States of America; University College London, UNITED KINGDOM

## Abstract

Prospective memory tasks are tasks that one must remember to perform in the future, such as keeping a dentist appointment or locking the door when leaving home. There has been little research to date on the question of what motivates real-life prospective memory tasks, and this is true both generally and within the subfield of aging and prospective memory. In the current study, we investigated whether the prospective memory tasks of younger and older adults were motivated by different personal goals and concerns, a question that has not been addressed in past research. Participants completed a questionnaire on current prospective memory tasks and the higher level goals and concerns that motivated these tasks. In general, younger and older adults reported prospective memory tasks motivated by different goals and concerns that reflected different social age systems or developmental tasks. Specifically, younger adults were more likely to report prospective memory tasks related to goals for education, profession, property, self, and leisure, and related to concerns about education and profession. In contrast, older adults were more likely to report prospective memory tasks related to concerns about world issues and war/terrorism. We also examined prospective memory task motivation more generally as approach motivation (goal-relatedness) and avoidance motivation (concern-relatedness). Both measures showed a gender by age group interaction. That is, older males showed especially low approach motivation and especially high avoidance motivation for their real-life prospective memory tasks. We suggest that a new approach to prospective memory research that incorporates motivational influences would enhance the ecological validity of prospective memory and aging research and may inform more effective memory interventions.

## Introduction

Prospective memory tasks refer to actions one wants to remember to perform in the future [1]. Examples of prospective memory tasks include remembering to take a blood pressure medication or sending a birthday card to a relative. Forgetting these tasks can have negative consequences for both younger and older adults.

A fair amount of research on prospective memory has been devoted to prospective memory and aging (e.g., for reviews see [2–4]). That is, researchers have examined how prospective memory is different, and similar, in early adulthood and later adulthood.

However, there is a major gap in research on prospective memory and aging. Motivational variables have not been sufficiently examined. In particular, researchers have neglected the role of goals and concerns. Goals are fundamental in modern conceptions of motivation and have been defined as desired future states [5–7]. The related construct of concerns refers to mental representations of future states that a person is afraid of or worried about [7]. Research on these two constructs—goals and concerns—is sometimes grouped together under the umbrella term “goals research,” with goals representing outcomes a person wants to achieve and concerns representing outcomes a person wants to avoid [8]. It is hard to overestimate the importance of goals in everyday life. In brief, goals drive behavior [9–11]. There is an extensive literature on personal goals [12], and even a sub-field focused on age-related differences in goals (e.g., [13, 14]), but there is still a need for research that specifically examines age-related differences in goals motivating real-life prospective memory tasks, the more specific activities we must remember to do each day.

Therefore, the general purpose of the present study was to help fill this gap by examining the goals and concerns that motivate real-life prospective memory tasks in younger and older adults. To accomplish this, we conducted a questionnaire study in which we asked younger and older adults to list their upcoming real-life prospective memory tasks and then to tell us what motivated these tasks, that is, the types of goals and concerns that motivated their tasks. With this method, we relied on the individuals to report on their motivations for their planned tasks. Our more specific purpose was to address three research questions. First, do younger and older adults differ in the types of goals and concerns that motivate their real-life prospective memory tasks? Second, are there gender differences in motivation for real-life prospective memory tasks or interactions between gender and age? Third, do younger and older adults show general differences in approach versus avoidance motivation for their prospective memory tasks?

### Past literature on prospective memory and aging: A predominantly cold cognitive approach

Past research on prospective memory and aging has predominantly taken a “cold cognitive” approach. That is, it has largely focused on cognitive functions or on task contexts that might explain differences in prospective memory performance between younger and older adults. This literature has produced a wealth of knowledge about prospective memory and aging (e.g., [2–4]). For instance, research has shown that significant rates of forgetting do occur in both younger and older adults, with older adults generally performing worse, although not always [15, 16]. In a meta-analytic review of prospective memory and aging, two key variables were identified that appeared to influence whether older and younger adults differed in prospective memory performance: 1) strategic/controlled processing requirements for the prospective memory task, and 2) task context [2]. Greater age-related deficits in remembering tend to occur with prospective memory tasks that have higher controlled processing requirements. For example, in a prospective memory task that entails trying to remember to spot target words on a screen, watching for a general category target (e.g., “animals”) requires more controlled processing than watching for a specific target (e.g., “dog”; e.g., [17]). Concerning task context/location effects, young adults usually outperform older adults in laboratory settings, which tend to be under highly controlled conditions (e.g., [18, 19]). However, on naturalistic tasks (e.g., telephoning the experimenter), older adults usually perform as well as, or even better than, young adults (e.g., [16, 20]). This pattern of differences can be summarized as the paradoxical age-related differences between naturalistic and laboratory prospective memory tasks [21].

Researchers have made progress in elucidating the causes of these paradoxical findings [4, 22]. For example, in a carefully controlled study, Gilbert [23] found that older adults were more likely to use an external reminder than younger adults were (i.e., more likely to offload the memory burden to an external visual cue). This strategy difference might partly explain why older adults can remember to perform intentions fairly well in their home setting or other naturalistic environments. However, in a diary study, Ihle and colleagues [24] found equal rates of reminder use for younger and older adults. They did, however, find an age-related benefit for performing everyday prospective memory tasks. This advantage in the older group was related to adopting a strategy to reprioritize initially planned intentions. Higher task importance was also related to better performance rates. Although lower stress predicted better prospective memory performance, the age-related benefit to performance was not explained by stress level. In a very recent study, Schnitzspahn and colleagues [22] examined a variety of types of prospective memory tasks in young and older adults (time-based and event based tasks; lab tasks, experimenter-assigned naturalistic tasks, and self-assigned naturalistic tasks). They found that the age-related differences in performance depended on both context (lab or non-lab) and type of cue. For example, age-related benefits outside of the lab were only found for naturalistic time-cued tasks, and not for self-assigned time-based tasks. In summary, past research suggests that the mechanisms that underlie the prospective memory and aging paradox may be numerous and complex.

Despite the research advances outlined above, we argue that the overall field of research on prospective memory and aging has emphasized a predominantly cold cognitive approach and this approach is limited for studying age effects, including those in the prospective memory and aging paradox. That is, considering motivational influences in more studies could provide more ecologically valid, and possibly more complete, explanations for how we remember to perform delayed intentions and how this changes in later years.

### Prospective memory and aging: A motivational-cognitive approach

People do not form prospective memory tasks in a vacuum. Rather, they are motivated to form certain intentions, and some prospective memory tasks are viewed as especially important [25, 26]. Yet this hot motivational context of prospective memory is often neglected in empirical investigations and theories of prospective memory and aging. Recently, several investigators have called for more research on motivation as one way to improve the ecological validity of studies of prospective memory and aging [4, 26, 27]. Motivation in prospective memory has been assessed in several studies that are high in ecological validity—studies that assessed real prospective memory tasks listed by participants. For example, in the diary study by Ihle and colleagues [24] described earlier, participants not only listed their real prospective memory tasks, they also rated their importance. Results showed that for both young and older adults, performance was higher for more important tasks, though task importance benefited young adults even more than older adults. Marsh and colleagues [28] had young adults list their upcoming prospective memory tasks for the week and rate their importance. Participants completed more important tasks at a higher rate than less important tasks. Recently, Schnitzspahn and colleagues [25] examined real prospective memory tasks in young and older adults using a 30-day diary task. They found that age-related differences in performance varied by prospective memory task domain (social, work-related, health-related, organizational and housekeeping, or leisure time). Older adults remembered to perform tasks more than younger adults in two of the five task domains (social and health). Interestingly, one of these two domains, social, was the only domain where older adults rated their tasks as higher in importance than young adults. Therefore, task importance may have benefited older adults’ remembering, but only for tasks that have social consequences. In sum, past research has shown that task importance matters for both young and older adults, but these benefits might interact with age and with different life domains for the tasks. Building on this past research on task importance effects, we sought to examine age differences in higher goals and concerns that motivate everyday prospective tasks.

### Why might goals and concerns be important in understanding prospective memory and aging?

No past studies have examined how the goals and concerns motivating everyday prospective memory tasks might be different for younger and older adults. For example, the motivation behind remembering to go swimming on a given morning might be very different for a 20 year old woman and a 70 year old woman. The 20 year old might be motivated to remember because she is motivated by goals for self-improvement such as being more physically attractive or experiencing the improved mood that results from exercise. In contrast, the 70 year old might be motivated to remember because she is motivated by goals for improving her health such as management of her diabetes. We argue that motivational differences such as these are important in understanding the possible influence of aging on prospective memory performance. This argument is derived from two literatures: a literature supporting the idea that intentions (prospective memory tasks) are linked in memory to the goals they fulfill and a literature supporting the idea that higher-order goals and concerns of older adults differ systematically from those of younger adults.

First, we have proposed that prospective memory tasks are linked in memory to higher goals and concerns [26]. Other prospective memory researchers have also argued that real-life prospective memory tasks are in support of higher goals and so prospective memory research needs to reflect these relationships more [27]. Although both prospective memory tasks and goals involve motivated action and are important in self-regulation, there are important differences between these two constructs. In brief, goals are positive outcomes a person is trying to achieve or negative outcomes a person is trying to avoid. For example, Emmons [29] defines goals as “desired states that people seek to obtain, maintain, or avoid” (p. 314). He conceptualizes goals as “personal strivings,” defined as what someone is typically or characteristically trying to do, such as “be physically attractive” or “avoid conflicts with people.” Other goal researchers conceptualize goals in different ways, such as ideal selves (E.g., [30]), or life tasks (e.g., getting good grades) [31]. However, all goal definitions refer to outcomes to be achieved or avoided. In contrast to goals, prospective memory tasks are specific, delayed responses to a cue. The cue can be an event (e.g., when I see Tom), a time (e.g., at 6 p.m.), or an activity (e.g., when I start making dinner) [32]. Gollwitzer and Cohen [27] illustrated the difference between a goal and a prospective memory task with the example of a real airplane crash in 1988, Delta Flight 1141 out of Dallas, Texas. The plane crashed during take-off, killing 14 people on board. Gollwitzer and Cohen note that all crew members and air traffic controllers had the same goal: a safe take-off. However, the flight crew had apparently failed to perform an important prospective memory task: setting the flaps and slats to the correct configuration for take-off.

There is a rich history in goals research for hierarchical descriptions for the psychology of action, with goals represented at the highest level and concrete actions represented at the lowest level [12, 33, 34, 35, 36]. For example, in goal systems theory, goal representations are seen as part of a larger cognitive/motivational network (e.g., [33, 36]). Goals (personal goals), at the highest level, are linked to or possess associative connections to lower-level “means” or subordinate activities that support the goals. For example, an older man might have the goal of maintaining healthy blood pressure levels. This goal representation might be linked to representations for subordinate activities such as “exercising” and “taking blood pressure medication.” Personal concerns are assumed to function like goals but instead represent outcomes to be avoided (e.g., [7, 37]). There has been accumulating evidence that these various entities in goal networks are associatively linked (e.g., [38]). For example, superordinate goals have shown “top-down” activation, boosting performance for related intentions [39].

We extended hierarchical goal models and tailored them to apply to prospective memory tasks specifically by presenting a motivational-cognitive model of prospective memory [26]. According to this model, prospective memory tasks are considered highly specific intentions, represented at a lower level, that is, a level subordinate to representations for “means/activities.” The construct of prospective memory refers to a special subset of actions. That is, not all events or actions in daily life are prospective memory tasks. Prospective memory tasks involve bringing back to consciousness a plan that was formed in the past, and doing so at the correct time and place, and in response to a cue to act [40]. Accordingly, prospective remembering is sometimes called realizing delayed intentions [41]. In contrast, everyday actions can include other things such as reflexive actions, vigilance tasks (which keep the intention in consciousness), and responses to other people (e.g., speaking during a conversation or acting when directed by another person).

Consider an example to illustrate the links between prospective memory tasks and higher goals in our motivational-cognitive prospective memory model. The previously described older man might set the prospective memory task “buy walking shoes at the mall tomorrow,” which supports the activity of exercising, which supports his goal of maintaining healthy blood pressure levels. In this model, all prospective memory tasks are not alike; those that possess associative links to important goals benefit from these connections in terms of enhanced automatic and controlled processes and strategies [26]. The model also tailors hierarchical goal models to prospective memory by representing a prospective memory task as a complex, multi-phase process (e.g., [41, 32, 42]). That is, following past research and theory on the phases of a prospective memory task, it is parsed into separate phases, such as intention formation, intention retention, and execution. Motivational influences, including goal activation, can theoretically impact any phase of the prospective memory process.

Recent studies have provided evidence supporting key predictions from this model. For example, Cook, Rummel, and Dummel [43] showed that framing a goal as either a gain or a loss (vs. no goal) caused better performance for a prospective memory task supporting the goal. Penningroth and Scott [44] found that prospective memory tasks linked to goals (i.e., tasks that were more important) spurred greater use of memory strategies.

Second, the higher-order goals and concerns of older adults and younger adults are thought to differ in important and systematic ways (e.g., [7, 13, 14, 37, 45]). These distinctions are common in models of adult development. For example, Helsen, Kwan, John, and Jones [46] put this strongly by stating, “theories of adult development all agree that adulthood is a time of important changes in goals, resources, and coping” (p. 287). More specifically, the goals and concerns of older and younger adults are thought to differ in a way that reflects differences in their developmental tasks [45, 47]. Havighurst [45] described developmental tasks as tasks that individuals in a culture are expected to master during specific life periods. For example, the developmental tasks of early adulthood in America include selecting a mate and getting started in an occupation. In contrast, the developmental tasks of later adulthood include adjusting to decreasing physical strength and health. Saajanaho and colleagues[48] observed changes in the personal goals reported by older women in Finland across an eight-year longitudinal study. For example, goals for exercise and cultural activities decreased, and the authors concluded that reduced mobility (e.g., walking ability) may have driven the shifting of personal goals in these women.

There is other empirical evidence to support these theorized age-related differences in goals. For example, Cross and Markus [37] examined “possible selves” in four age groups, including young adulthood (age 18–24), maturity (age 25–39), middle age (age 40–59), and older adulthood (age 60–86). Participants listed possible selves that were hoped for (analogous to goals) or feared (analogous to concerns). Results showed systematic differences between age groups in the categories of possible selves. Specifically, the four age groups reported possible selves that aligned fairly well with socially relevant “developmental tasks” (e.g., [45]). Cross and Markus [37] found that the hoped for possible selves of the youngest group were categorized more often as related to family (e.g., marrying the right person). The hoped for possible selves of the oldest group were more focused on maintaining health/physical abilities (e.g., being in good health and vigor) and less focused on occupational aspirations than other groups. Similarly, the feared possible selves for the three younger groups were more focused on family issues and education/aspirations than the oldest group. The feared possible selves for the oldest group were distinguished by a focus on concerns about health/physical abilities.

Nurmi [7] examined age differences in stated goals and concerns across five adult age groups in a Finnish sample (ranging from 19–64 years), expecting to find age differences that reflected the relevant social age systems [47], such as the age-graded developmental tasks described by Havighurst [45]. In short, Nurmi [7] found that adults in different age groups did report goals and concerns from dissimilar content categories. Further, the results supported the conclusion that the age differences in goals and concerns reflected differences in the groups’ developmental tasks. That is, the pattern for young adults showed frequent reporting of goals for education, family/marriage, and property, and frequent reporting of concerns about themselves and their friends. In contrast, the pattern for older adults showed frequent reporting of goals for their health, leisure activities, the world, and retirement, and concerns for their health and war.

In summary, we argue that prospective memory performance occurs in a hot motivational context, namely through connections to larger hierarchically structured goal/concern knowledge networks. There is abundant evidence that personal goals and concerns drive everyday behaviors. Also, the goals and concerns of older adults differ from those of young adults. Our focus in the current study was on the specific set of goals and concerns linked to people’s real-life planned actions—their prospective memory tasks. Would these motivational forces differ for young and older adults? If so, how?

### Present study: Age and gender-related differences in motivation for real prospective memory tasks

We had three major research questions. The first research question was *Do younger and older adults differ in the types of goals and concerns that motivate their real-life prospective memory tasks*? For the hypotheses relevant to this question, we predicted age-related differences that would generally reflect socially ascribed developmental tasks (e.g., [45]) or “social age systems” [47]. That is, we expected to find the same age-related pattern of motivational influences that has been reported in the more general goal literature (e.g., [7, 37, 45]). However, whereas past researchers have asked about all goals, we asked participants only about the goals that motivated their real prospective memory tasks. Specifically, the first hypothesis was that compared to older adults, younger adults would have more tasks motivated by goals and concerns in domains relevant to the developmental tasks of early adulthood. Based on past research and theorizing (e.g., [7, 37, 45]), we predicted that such goals would include those related to education, profession, marriage, children, property/possessions, self/personal growth, and friendship. The second hypothesis was that compared to younger adults, older adults would have more tasks motivated by goals and concerns in domains relevant to the developmental tasks of later adulthood. Based on past research and theorizing (e.g., [7, 37, 45]), we predicted that such goals would include those related to health, leisure, world issues, retirement, and war/terrorism.

Our second research question was *Does motivation for real-life prospective memory tasks show gender differences or interactions of gender and age*? Research on gender differences in prospective memory is scarce. A few studies have found that females show superior performance in remembering [49, 50] and use prospective memory strategies (e.g., written reminders) more [44]. However, we found no past studies on gender differences in goals and concerns linked to prospective memory tasks. Several theoretical descriptions of social age systems or social roles do emphasize that developmental life patterns can be very different for males and females (e.g., [45, 51, 52]), and so different domains of motivation might be predicted. For example, in their twenties, females might be more likely than males to postpone occupational goals in order to raise children. Yet, the evidence for consistent gender differences in life goals has been mixed. For example, Nurmi [7] did not find the predicted pattern of more family-oriented goals and concerns for females and more profession-oriented goals and concerns for males. In contrast, Evans and Diekman [53] found that in their young adult sample, females rated distant goals for caregiving (e.g., attending field trips) as more important than males, who rated status goals higher (e.g., running a company or organization). Given the mixed evidence on gender differences in past studies and the absence of research addressing motivation for prospective memory tasks specifically, our approach in the current study was exploratory. That is, we had no specific hypotheses for this second research question.

Finally, our third research question was *Do younger and older adults differ in approach and avoidance motivation for their real prospective memory tasks*? Whereas approach motivation focuses on attaining outcomes, avoidance motivation focuses on avoiding outcomes. For instance, a person might meet with an acquaintance because he or she wants to develop a friendship (approach) or because of concern about loneliness (avoidance; [10]).

In past research on approach and avoidance motivation, these constructs have been measured in different ways. For example, Cross and Markus [37] had participants list hoped-for possible selves, which seem to align with approach goals. Example definitions for measures for hoped-for possible selves included “what we hope or wish we could be like…for example ‘vacationing in Florida” [37] (p. 235). In contrast, feared possible selves seem to align with avoidance goals. Example definitions for measuring feared possible selves included “the ways we are afraid we might turn out to be…possible selves that are feared or dreaded…like ‘being in poor health’” [37] (p. 235). In the specific domain of achievement motivation in a class, the Achievement Goal Questionnaire [54] exemplifies a method for measuring approach and avoidance motivation as goals. Participants use a 1–7 scale to rate the extent to which each of 12 items is true for them. Six items measure approach goal motivation (e.g., I want to learn as much as possible from this class; My goal in this class is to get a better grade than most of the other students) and six items measure avoidance goal motivation (e.g., My fear of performing poorly in this class is often what motivates me; I am often concerned that I may not learn all that there is to learn in this class). In the current study, we measured approach motivation as greater motivation from goals to be attained (defined for participants as personal goals, that is, current goals, hopes, plans, and dreams). We measured avoidance motivation as greater motivation from concerns (defined as things you are currently afraid of or worried about).

People differ in their characteristic motivational orientation, that is, their tendencies to think in terms of approach or avoidance (e.g., [55, 56]). In general, an avoidance orientation has been shown to be less optimal [10]. For instance, avoidance motivation promotes lower efficacy beliefs for goal-related behaviors and lower goal commitment and also leads to worry and distraction that undermines goal-related intrinsic motivation and performance [10, 57]. Further, in one experiment, avoidance motivation caused impaired memory performance [58].

We found no past research on age-related differences in approach and avoidance motivation linked to prospective memory tasks. However, researchers have examined whether older and younger adults differ in approach and avoidance more generally, that is, by comparing higher-level goals that are stated or can be inferred. Empirical evidence bearing on this question has been mixed, but there are theoretical and empirical reasons to expect specific age group differences. For example, according to selection, optimization, and compensation theory (e.g., [13, 59]), as individuals age they tend to have relatively fewer personal goals motivated by gains or improvements (e.g., learning about other cultures) and more concerns motivated by loss prevention and maintenance of current functioning (e.g., not getting sick). Supporting this theory, these age-related differences have been found in empirical studies (e.g., [11, 14, 60, 61]). However, Cross and Markus [37] found that in a sample of 18 to 86 year olds, both the number of generated goals (i.e., hoped for possible selves) and concerns (i.e., feared possible selves) decreased with age. In contrast, in a sample of 19 to 64 year olds, Nurmi [7] found that age was correlated with a decrease in the number of goals listed but age was not related to the number of concerns listed. In summary, past research has not consistently supported the claim that older adults are more motivated by avoidance of bad outcomes and younger adults are more motivated by aspirations for good outcomes, but the bulk of the extant literature supports this view.

In the current study, we were not interested in all goals people held, but instead in the goals and concerns that were specifically linked to current prospective memory tasks. Our third hypothesis was that compared to older adults, younger adults would have more tasks motivated by goals. This finding would reflect stronger approach motivation for everyday prospective memory tasks in younger adults. The past evidence is less clear when it comes to informing a prediction about whether younger and older adults differ in tasks motivated by concerns (avoidance motivation). However, for the fourth hypothesis, we tentatively predicted that compared to younger adults, older adults would have more tasks motivated by concerns. This finding would reflect stronger avoidance motivation for everyday prospective memory tasks in older adults.

In brief, our overall aim was to fill gaps in research on prospective memory and aging. No previous studies have examined whether the personal goals and concerns related to everyday prospective memory tasks differ across age groups. We used a questionnaire that asked younger and older participants to list five of their current prospective memory tasks. For each listed task, they then indicated the types of goals and concerns supported by the task (i.e., which of 15 goal and concern content categories (e.g., your health) the task supported).

## Materials and method

### Participants

We estimated the sample size needed from two past studies. A study that tested how much daily events were related to seven goal categories in young adults found that the effect sizes for the goal relatedness dependent variables ranged from *d* = .78 to *d* = 1.38 [31]. Based on this study, our a priori power analysis for alpha = .05 and power = .80 for our planned independent groups *t* tests (young vs. old) suggested a minimum sample size between 17 and 27. Another past study included comparisons of young adults (age 18–24) to older adults (age 60+) on frequencies of different types of future goals listed (hoped for selves and feared selves) [37]. Most statistical values were not reported, and so we could not calculate effect sizes. However, significant age-related differences were found for multiple categories of goals using ANOVA with a sample size of 100. In sum, based on these past two studies, our initial goal for sample size was 100. We report post-hoc power calculations (1-β) for all analyses relevant to the hypotheses in the results section.

Eligibility for the study was advertised as being between age 18–30 or age 60–85 and being a native English speaker. There were 89 participants (56 younger and 33 older adults) in the final sample. The original sample contained 104 participants, but data for 15 participants (11 younger and 4 older adults) were dropped because they did not follow instructions or they did not finish the survey (one of the 11 participants in the younger group who was excluded for not following instructions was also too old (34 years old)). All of the participants were either native English speakers or attending a college where instruction was in English (two of the younger adults). Our exclusion criteria also included being in poor overall health (indicated by a self-reported rating of less than “fair” overall health on the survey) or self-reported living in a nursing home or assisted living facility. However, no participants reported these answers and so none were excluded for these reasons. This was not surprising because the older adults were recruited at a community activity center for seniors and probably represented a relatively healthy, socially active population. Participants were categorized into age groups and gender groups based on self-reported information. That is, participants reported their age and checked whether they were female or male, but these answers were free-response items and so they could be left blank or another option could be filled in (for gender). However, none of the participants left these blank or reported a different gender identity. The Institutional Review Board at the University of Wyoming approved the procedure for this study. Demographic information is displayed in Table 1.

**Table 1:** Demographic variables.

	Young	Young	All	Older	Older	All
	Males	Females	Young	Males	Females	Older
*N*	22	34	56	12	21	33
% Female			60.7			63.6
Age range	18–26	18–29	18–29	66–85	65–85	65–85
Age: Mean	20.23	19.79	19.96	76.25	74.53	75.19
Age: SD	2.67	2.76	2.71	5.94	6.11	6.01
Education: Mean	13.59	14.21	13.96	15.17	13.67	14.21
Education: SD	1.50	1.90	1.77	2.92	2.63	2.79
Health: Mean	4.14	4.03	4.07	4.00	3.95	3.97
Health: SD	0.35	0.67	0.57	0.85	0.62	0.71
% Married	4.5	17.6	12.5	58.3	42.9	48.5
% Divorced or widowed	0	0	0	33.3	47.6	42.4
% Never married	81.8	76.5	78.6	0	0	0
% No marital status given	13.6	5.9	8.9	8.3	9.5	9.1

*Note*. Age was measured in years. Education was measured as reported number of years of education. Health was measured as a rating of perceived current health (1 = very poor; 5 = very good). SD = Standard deviation. See text for breakdowns for living arrangements/housing.

Young adults were recruited from two populations: 32 undergraduate students who participated as partial fulfillment of course requirements and 24 non-students who were recruited through advertisements and referrals from students and were paid $10 (USD). Self-reported living arrangements/housing showed that for the 56 young adults, 55.4% lived in their own home or apartment, 39.3% lived in a dorm or fraternity, and 5.4% lived with a family member. For the young male subgroup (n = 22), 54.5% lived in their own home or apartment, 36.4% lived in a dorm or fraternity, and 9.1% lived with a family member. For the young female subgroup (n = 34), 55.9% lived in their own home or apartment, 41.2% lived in a dorm or fraternity, and 2.9% lived with a family member. The young adults completed the questionnaire in a college classroom or the public library.

All older adults were recruited at a community senior center that provided recreational and health services for senior citizens. They completed the questionnaire during or after lunch at the senior center and were paid $5 (USD). Most of the older adults were retired (78.8%). For the 33 older adults, 90.9% lived in their own home or apartment, 3% lived with a family member, and 6.1% did not report their living arrangements. For the older male subgroup (n = 12), 100% lived in their own home or apartment. For the older female subgroup (n = 21), 85.7% lived in their own home or apartment, 4.8% lived with a family member, and 9.5% did not report their living arrangements.

### Materials and procedure

All participants completed a questionnaire in a session lasting no more than 30 min. Data were collected in October and November of 2005. An experimenter administered the paper questionnaire to groups that ranged in size from 3 to 33. The cover page was labeled “SURVEY ON REAL-LIFE ACTIVITIES” and also listed “Instructions for the entire survey.” These included instructions to read all instructions carefully, not look ahead in the survey, and ask the experimenter if you have questions. Three sections were relevant to the current study: a listing of real-life prospective memory tasks, a form for indicating whether the listed prospective memory tasks were related to goal/concern categories, and a demographics survey.

#### Listing of real-life prospective memory tasks

Instructions in the questionnaire were stated as follows.

This section asks you about remembering to do something. This includes appointments, tasks, and other things you want to remember to do. For example, if you intend to see the movie “Chicken Little” this Sunday, write “See the movie ‘Chicken Little’ Sunday.” In the table below, please list 5 specific examples of things you want to remember to do, from your own life. Don’t list habits or activities that you would do automatically like sleeping, having dinner, or brushing your teeth. Use complete phrases so it’s clear what the task is (don’t just summarize the task in a single word).

The table for listing the five tasks contained five numbered slots, with the heading: *EXAMPLES of things you want to remember to do*. These instructions were adapted from those we had used in a past study [62], which were adapted from instructions used by Maylor, Chater, and Brown [63]. However, in the current study we also added the instruction to exclude habits or activities that you would do automatically like sleeping, having dinner, or brushing your teeth, which we adapted from a study by Freeman and Ellis [64]. Most participants listed five prospective memory tasks, as requested, but three participants (all older) listed less than five.

We decided on having participants list five prospective memory tasks based on past published research from our lab and others [27, 62, 65] and from pilot testing of the key measures, which showed that listing 12 tasks and relating them to goals and concerns was too onerous for participants, resulting in high rates of non-completion. However, limiting the list to five tasks probably means these tasks are relatively high in importance when compared to all tasks a person intends to do.

#### Rating if prospective memory tasks are related to goal or concern categories

The questionnaire directly measured the goal-relatedness and concern-relatedness of participants’ real tasks. That is, participants reported whether each of their five prospective memory tasks listed was directly related to a personal goal or concern. More specifically, participants filled in a form that contained five rows and 15 columns. They first recopied the five prospective memory tasks from the earlier section into the five rows of the left-hand column of the table. Across the top of this form appeared 15 goal/concern category labels: *your profession/occupation*, *your property/possessions*, *your health*, *the health of others*, *your children’s lives*, *marriage/relatives*, *your education*, *travel*, *leisure activities*, *retirement*, *war/terrorism*, *world issues*, *friendship*, *self/personal growth*, and *other*. These goal/concern categories were adapted from those used by Nurmi and colleagues [7, 66]. That is, we assessed the same content domains as Nurmi and colleagues, but we asked participants to tell us the content domain for goals and concerns that motivated a set of prospective memory tasks they listed whereas participants in Nurmi’s studies listed their general goals and concerns. Thus, instructions directed respondents to indicate whether each of their prospective memory tasks was related to each of the 15 goal/concern categories. Specifically, the questionnaire instructions stated:

We would like to know if any of the five task examples you listed are related to your personal goals or concerns. Goals are defined as your current goals, hopes, plans, and dreams. Concerns are defined as things you are currently afraid of or worried about. Read the first example you copied into the table below. Judge whether it’s directly related to your personal goals or concerns in each category. That is, first, consider whether the example you wrote is related to a goal or a concern you have for your profession or occupation. If it is related to that type of goal, circle “goal” in that column. If it is related to that type of concern, circle “concern” in that column. Then, do the same thing for the next goal/concern category “property/possessions.” Continue across the row, doing the same for all categories…Therefore, for each example you listed, you will circle between 0 and 15 related goal categories, and between 0 and 15 related concern categories. You may circle both “goal” and “concern” for the same category.

#### Assessing demographic variables

Finally, the last section of the questionnaire assessed demographic variables including gender, age, education, marital status, current living situation (e.g., in own home/apartment, in the college dorms), and perceived overall health.

## Results

An alpha level of .05 was used for all analyses. To reduce the experiment-wise Type I error rate, two-tailed significance levels are reported for all statistical analyses, even when hypotheses were directional.

Examples of prospective memory tasks participants listed, along with related goal and concern categories, appear in Table 2.

**Table 2:** Examples of prospective memory tasks listed by participants, with the motivating goal or concern categories.

Example of Task	Related to Goal or Concern?	Goal or Concern Category the Task is Related to
*Work my second job on Sunday*	Goal	Profession
*Take car for lube job*	Concern	Property
*Doctor’s appointment tomorrow*	Goal	Own health
*Keeping husband’s doctor’s appointment*	Concern	Others’ health
*Go to neighbor’s so our children can play*	Goal	Children
*Bring my husband lunch tomorrow*	Goal	Marriage/relatives
*Set alarm for Bible study Monday*	Goal	Education
*Get airline ticket for Thanksgiving*	Goal	Travel
*Practice the drums*	Goal	Leisure
*Complete the reservations in Arizona*	Concern	Retirement
*Call a friend*	Concern	War/terrorism
*Give a compliment to a stranger every day*	Goal	World issues
*Call friend I haven’t talked to all summer*	Goal	Friendship
*Work out tonight*	Goal	Self/growth
*Take puppy in to get shots*	Concern	Other

### Preliminary analyses

Before running analyses, we examined whether the younger and older groups differed on gender composition, perceived overall health, or education (see Table 1; note that two older adults did not report perceived health). There were no differences on the three demographic variables: gender composition, χ^2^(1) = 0.08, *p* = .784, phi = .030; perceived health, *t*(85) = 0.75, *p* = .457, η^2^ = .007; or years of education, *t*(87) = 0.51, *p* = .609, η^2^ = .003. We also tested for gender by age group interaction effects in education and perceived health (see Table 1). These variables did not interact for perceived health (*F* < 1), but there was an interaction for education level, *F*(1,85) = 4.70, *p* = .034, η_p_^2^ = .052. As can be seen in Table 1, there was a pattern showing the highest education level in older males, then younger females, then older females and younger males. However, post-hoc tests revealed no significant differences between groups.

We also checked the listed prospective memory tasks to see how many were clearly prospective memory tasks. Thus, the first author coded all listed tasks into one of five categories. The first two categories were both types of prospective memory tasks, defined by applying the definitions in our instructions to participants and also by the definitions stated here, earlier, in the introduction (e.g., intended, specific actions that were previously planned to be executed in response to an event cue, time cue, or activity cue). The first category, episodic prospective memory tasks, additionally referred to tasks that occur infrequently or not on a regular schedule (e.g., send birthday present to sister). The second category, habitual prospective memory tasks, were defined as occurring more frequently and on a regular schedule (e.g., turn the heat down at night) [32, 40]. Coding for these two prospective memory categories was done conservatively. That is, the coder had to be fairly certain that the tasks fit, otherwise they were coded as one of the following three categories. The third category was goals, defined by applying the definitions in our instructions to participants and also by the definitions stated here, earlier, in the introduction (e.g., states that people seek to achieve or avoid), such as set up a church Sunday school. The fourth category was vague or unclear tasks. These could not be categorized as prospective memory tasks with high certainty: they were possibly prospective memory tasks, but were possibly goals (e.g., sell the other car). The fifth category was retrospective memory tasks, defined as intentions to recall information from the past (e.g., phone numbers (mine)).

Results from the five-category coding showed that 91% of listed tasks were clearly a type of prospective memory task (specifically, 55% were episodic prospective memory tasks and 36% were habitual prospective memory tasks). Goals constituted 6% of the listed tasks, vague/unclear tasks constituted 2% of the listed tasks, and retrospective memory tasks constituted 1% of the listed tasks. In sum, using conservative criteria, we concluded that with a few exceptions, participants understood and followed the instructions to list prospective memory tasks.

### Do younger and older adults differ in the types of goals and concerns that motivate their real prospective memory tasks?

The first hypothesis was that younger adults would have more prospective memory tasks motivated by goals and concerns in domains associated with the developmental tasks of early adulthood (i.e., education, profession, marriage, children, property/possessions, self/personal growth, and friendship). The second hypothesis was that older adults, in contrast, would have more prospective memory tasks motivated by goals and concerns in domains associated with the developmental tasks of later adulthood (i.e., health, leisure, world issues, retirement, and war/terrorism).

We examined prospective memory tasks related to goals separately from those related to concerns. For each of the 14 content categories (omitting the rarely used “other” category), participants were categorized as either reporting a task related to that goal category (or concern category) or not. For example, participants were categorized as having a prospective memory task that supported their education goals if they reported that any of their prospective memory tasks were related to that goal category. We did not use the percent of category-relevant tasks as the dependent variable because these distributions were highly skewed. Therefore, chi-square tests were used to compare the younger and older groups.

#### Age group differences in goal categories related to prospective memory tasks

Table 3 shows the percentages of each age group that reported prospective memory tasks related to each of the 14 goal categories. Of the 14 goal categories, five showed differences between younger and older adults. For all five, younger adults were more likely than older adults to report that they had current prospective memory tasks related to the category. Specifically, younger adults were more likely to report current tasks related to their goals for education, *p* < .001,for profession, *p* = .007, for property, *p* = .022, for self/personal growth, *p* = .040, and for leisure, *p* = .035.

**Table 3:** Percentage of sample or age group that reported prospective memory tasks related to specific goal categories.

Goal Category PM Tasks are Related to		Age Group				
	Overall Sample	Young	Older	χ^2^ (Young vs. Older)	Phi	Power (1-β)
Leisure	53.9	62.5	39.4	4.46*	.224	.561
Friendship	52.8	58.9	42.4	2.27	.160	.326
Self/growth	50.6	58.9	36.4	4.23*	.218	.515
Own health	48.3	42.9	57.6	1.80	.142	.268
Education	46.1	64.3	15.2	20.18***	.476	.994
Property	46.1	55.4	30.3	5.25 *	.243	.630
Profession	36.0	46.4	18.2	7.20**	.284	.764
Marriage/relatives	27.0	28.6	24.2	0.20	.047	.073
Others’ health	23.6	23.2	24.2	0.01	.012	.052
Travel	22.5	23.2	21.2	0.05	.023	.055
Children	18.0	16.1	21.2	0.37	.065	.094
Retirement	14.6	16.1	12.1	0.26	.054	.080
World issues	7.9	5.4	12.1	1.31	.121	.208
War/terrorism	4.5	1.8	9.1	2.58	.170	.361

*p < .05; **p < .01; ***p < .001. Categories are ordered from the most commonly reported (overall) to the least commonly reported. PM = Prospective memory.

#### Age group differences in concern categories related to prospective memory tasks

Table 4 shows the percentages of each age group that reported prospective memory tasks related to each of the 14 concern categories. Of the 14 concern categories, four showed differences between younger and older adults. Younger adults were more likely than older adults to report tasks that were related to their concerns for education, *p* < .001, and for profession, *p* = .017. In contrast, older adults were more likely than younger adults to report tasks that were related to their concerns for world issues, *p* = .022, and for war/terrorism, *p* = .002.

**Table 4:** Percentage of sample or age group that reported prospective memory tasks related to specific concern categories.

Concern Category PM Tasks are Related to		Age Group				
	Overall Sample	Young	Older	χ^2^ (Young vs. Older)	Phi	Power (1-β)
Property	55.1	53.6	57.6	0.14	.039	.066
Own health	40.4	39.3	42.4	0.09	.031	.060
Profession	33.7	42.9	18.2	5.66*	.252	.662
Education	31.5	48.2	3.0	19.66***	.470	.993
Leisure	29.2	26.8	33.3	0.43	.070	.101
Others’ health	28.1	28.6	27.3	0.02	.014	.052
Friendship	24.7	28.6	18.2	1.20	.116	.195
Self/growth	24.7	26.8	21.2	0.35	.062	.090
Travel	20.2	23.2	15.2	0.84	.097	.150
Children	16.9	14.3	21.2	0.71	.089	.134
Retirement	16.9	14.3	21.2	0.71	.089	.134
Marriage/relatives	15.7	19.6	9.1	1.74	.140	.262
World issues	11.2	5.4	21.2	5.23*	.242	.627
War/terrorism	9.0	1.8	21.2	9.58**	.328	.872

*p < .05; **p < .01; ***p < .001. Categories are ordered from the most commonly reported (overall) to the least commonly reported. PM = Prospective memory.

### Gender differences

We also tested for gender differences in goals and concerns related to prospective memory tasks. For goal relatedness, two categories showed a gender difference. More females (54.5%) than males (32.4%) reported tasks related to goals for education, χ^2^(1) = 4.12, *p* = .041, phi = .215, 1-β = .527. More females (63.6%) than males (29.4%) reported tasks related to goals for self/personal growth, χ^2^(1) = 9.85, *p* = .002, phi = .333, 1-β = .881. For concern relatedness, three categories showed a gender difference. More females (41.8%) than males (20.6%) reported tasks related to concerns for education, χ^2^(1) = 4.24, *p* = .040, phi = .218, 1-β = .539. In contrast, more males (73.5%) than females (43.6%) reported tasks related to concerns for property, χ^2^(1) = 7.59, *p* = .006, phi = .292, 1-β = .787; and more males (38.2%) than females (9.1%) reported tasks related to concerns for travel, χ^2^(1) = 11.06, *p* = .001, phi = .353, 1-β = .915.

Given these gender differences, we also investigated whether both males and females showed the pattern of age-group effects found in the larger sample. Due to the small cell sizes for gender groups within age groups (see Table 1), the data violated the assumptions of the chi-square test and we were only able to examine descriptive patterns. However, the same patterns were seen in each gender group. That is, the age-group differences found in the larger sample were mirrored in both males and females. Therefore, we interpret these results as evidence that age affects the goals and concerns motivating prospective memory, and the pattern of these effects in the overall sample was also seen in both the male and female subgroups.

### Do younger and older adults differ in approach and avoidance motivation for their prospective memory tasks?

The third hypothesis was that, compared to older adults, young adults would generally show more approach motivation, that is, have more prospective memory tasks motivated by *goals*. We conducted a 2 (gender) x 2 (age group) between-groups ANOVA with the percentage of listed prospective memory tasks related to goals (i.e., related to goals in one or more goal categories) as the dependent variable. The predicted main effect of age group was marginally significant, with a trend for younger adults reporting more prospective memory tasks motivated by goals (*M* = 76.1%, *SD* = 27.6) than older adults did (*M* = 67.3%, *SD* = 33.0), *F*(1,85) = 3.74, *p* = .056, η_p_^2^ = .042, 1-β = .481. There was also a significant effect of gender, with females reporting more prospective memory tasks motivated by goals (*M* = 77.5%, *SD* = 25.8) than males did (*M* = 65.3%, *SD* = 34.6), *F*(1,85) = 6.15, *p* = .015, η_p_^2^ = .067, 1-β = .689. However, there was also a gender by age group interaction, *F*(1, 85) = 4.38, *p* = .039, η_p_^2^ = .049, 1-β = .544 . Post hoc tests showed that for younger adults, both females and males reported a high percentage of prospective memory tasks motivated by personal goals (younger females *M* = 77.1%, *SD* = 26.1; younger males *M* = 74.6%, *SD* = 30.4), and these percentages were equivalent, *t*(54) = 0.33, *p* = .743, η_p_^2^ = .002, 1-β = .076. In contrast, for older adults, although the older female subgroup also reported a high percentage of prospective memory tasks motivated by goals (*M* = 78.1%, *SD* = 25.8), the percentage was much lower for the older male subgroup (*M* = 48.3%, *SD* = 36.6), and this difference was significant, *t*(31) = 2.73, *p* = .010, η_p_^2^ = .194, 1-β = .978. Thus, older males seemed to differ from the other three subgroups in showing a relatively low number of prospective memory tasks with approach motivation, that is, motivated by personal goals. In fact, the percentage for the older males was significantly lower than all other subgroups, that is, also lower than younger males, *t*(32) = 2.24, *p* = .032, η_p_^2^ = .135, 1-β = .905, and younger females, *t*(44) = 2.94, *p* = .005, η_p_^2^ = .164, 1-β = .997.

Our fourth, more tentative, hypothesis was that compared to younger adults, older adults would show more avoidance motivation, that is, have more prospective memory tasks motivated by *concerns*. We conducted another 2 (gender) x 2 (age group) between-groups ANOVA, but for this hypothesis, the dependent variable was the percentage of listed prospective memory tasks related to concerns. There was no main effect of age group, with younger adults (*M* = 55.4%, *SD* = 32.6) and older adults (*M* = 53.9%, *SD* = 35.4) reporting the same percentage of prospective memory tasks motivated by concerns, *F*(1,85) = 0.15, *p* = .705, η_p_^2^ = .002, 1-β = .066. There was also no main effect of gender, with females (*M* = 52.0%, *SD* = 31.6) and males (*M* = 59.4%, *SD* = 36.3) reporting the same percentage of prospective memory tasks motivated by concerns, *F*(1,85) = 2.47, *p* = .119, η_p_^2^ = .028, 1-β = .343. However, there was an interaction between gender and age, *F*(1, 85) = 4.63, *p* = .034, η_p_^2^ = .052, 1-β = .567. Post hoc tests showed that for younger adults, females and males reported a moderate percentage of prospective memory tasks motivated by concerns (younger females *M* = 57.1%, *SD* = 29.2; younger males *M* = 52.7%, *SD* = 37.8), and these percentages were equivalent, *t*(54) = 0.48, *p* = .631, η_p_^2^ = .004, 1-β = .113. In contrast, for older adults, although the female subgroup also reported a moderate percentage of prospective memory tasks motivated by concerns (*M* = 43.8%, *SD* = 34.3), the percentage was much higher for the male subgroup (*M* = 71.7%, *SD* = 31.3), and this difference was significant, *t*(31) = 2.32, *p* = .027, η_p_^2^ = .147, 1-β = .919. Thus, older males again seemed to differ from the other three subgroups, this time showing a relatively high number of prospective memory tasks with avoidance motivation, that is, tasks motivated by concerns. However, additional post-hoc comparisons showed the older males’ percentage was not significantly different than that for the two younger subgroups, that is, the percentages for younger males *t*(32) = 1.48, *p* = .149, η_p_^2^ = .064, 1-β = .580, or younger females, *t*(44) = 1.46, *p* = .150, η_p_^2^ = .046, 1-β = .644.

## Discussion

To summarize, we found considerable support for our main argument that the underlying motivations for real-life prospective memory tasks for younger and older adults are different. Younger and older adults reported different goals and concerns motivating their real prospective memory tasks, with our findings largely conforming to adult developmental models of the unique challenges that these age groups confront (e.g., [45, 47]). We also found some support for the existence of gender differences in the domains of motivation for prospective memory tasks. Finally, age-related differences in approach and avoidance motivation for prospective memory tasks were found, but they depended on gender group. That is older men reported a lower percentage of prospective memory tasks motivated by goals and a higher percentage of tasks motivated by concerns, when compared to other subgroups. Thus, motivation for what we are trying to remember to do differs by age group, and by gender. We discuss these findings, and their implications, in detail below.

### Younger and older adults’ prospective memory tasks are motivated by different goals and concerns

We expected that younger adults would be more likely to have prospective memory tasks motivated by goals and concerns associated with the unique developmental challenges of early adulthood (e.g., [45, 47]). In fact, compared to older adults, younger adults were more likely to report that their prospective memory tasks were motivated by their personal goals for education, profession, property, self/personal growth, and leisure. Of these, only the leisure category was not predicted. For concern domains linked to prospective memory tasks, younger adults were more likely to report the domains of education and profession. In contrast, our specific predictions for older adults were not as consistently supported. We found only that their tasks were more likely to be motivated by concerns in two areas, when compared to younger adults: world issues and war/terrorism.

Although our study is the first to examine whether and how younger and older adults differ in the goals and concerns motivating their real-life prospective memory tasks, our findings are generally consistent with the larger goal literature demonstrating age-related differences in goals (e.g., [7, 37]). Our findings extend this literature to also specifically include differences in the types of goals and concerns motivating everyday prospective memory tasks.

Our findings also add to our broader understanding of basic differences in prospective memory for younger versus older adults. We know from past research (e.g., [2]) that prospective memory performance can differ for young and old. Specifically, cognitive variables and environment/context consistently affect whether younger and older adults show a difference. Our findings add to this literature, showing that younger and older adults also have different types of goals and concerns driving some of their real prospective memory tasks.

A number of authors have underscored the need for increasing the ecological validity of prospective memory research [4, 27]. Our focus on the motivating goals and concerns advances this aim. Further, our finding that younger and older adults have different motivations for their everyday prospective memory tasks presents a possible confound when comparing adults of different ages. For example, a researcher who wants to test the role of a cognitive variable (e.g., working memory capacity) in age-related prospective memory performance differences would be advised to reduce possible confounding motivational variables. Motivational variables could be held constant by ensuring the set of prospective memory tasks is equally related to the goals and concerns of both age groups (and ideally assessing goal/concern relatedness so that any pre-existing group differences could be statistically controlled).

The current results partially match those from some studies that have examined motivation in prospective memory for different age groups. For instance, we found that more young adults than older adults had prospective memory tasks motivated by goals for property/possessions. One might therefore predict that only younger adults would show a boost in prospective memory task performance when motivated by a monetary reward. This is exactly what was found in a study by Aberle, Rendell, Rose, McDaniel, and Kliegel [67]. Some of our results align with those from the 2016 study by Schnitzspahn and colleagues, described earlier [25]. In that study, independent raters sorted listed everyday prospective memory tasks into five categories (social, work, health, organization, and leisure) and found age-related differences in three categories: young adults had more intentions related to work than older adults did, and older adults had more intentions related to organization and leisure. Similarly, we found that young adults reported more intentions related to goals and concerns for work (profession) than older adults. Like Schnitzspahn and colleagues, we also found no age-related differences in intentions within the motivational categories of health or social domains (assessed as friendship or children or marriage/relatives in our study). We did not assess organizational domains of motivation in our study. Only one finding contrasted with the earlier study. Whereas Schnitzspahn and colleagues found that older adults reported *more* intentions related to leisure than younger adults, we found the opposite pattern: less intentions related to leisure goals compared to younger adults. We found no age-related differences in intentions related to *concerns* for leisure, though. Of course, there were several methodological differences between our study and that by Schnitzspahn and colleagues, but this makes the congruencies in some of the findings even more impressive. Taking the two studies together, these age-related motivational differences in prospective memory might be applied to memory improvement interventions that are tailored to the appropriate age group.

More broadly, the average rates of prospective memory tasks motivated by higher goals and concerns were fairly high (72.8% were goal-related; 54.8% were concern-related). This supports our contention [26] that goals and concerns are important in understanding prospective memory and age-related differences in prospective memory. However, it should be noted that the high rates of goal-relatedness and concern-relatedness for prospective memory tasks might be due to the fact that these tasks were highly accessible in memory (i.e., these were the five prospective memory tasks participants retrieved from memory). That is, according to the motivational-cognitive model of prospective memory [26], tasks with links to goals and concerns should be most accessible (retrievable) in memory. Therefore, we might expect lower rates of goal- and concern-relatedness if we asked participants to list more than five prospective memory tasks. This prediction would be an interesting one to test in future research.

### Gender differences

Our second research question addressed gender-related differences. Most importantly, we tested whether the age group differences in motivational content held for both males and females. Descriptive statistics suggested they did. That is, the patterns found in the larger sample appeared to be present in both the male and female subsamples (though, sample sizes for the four subgroups were too small to justify inferential statistical tests).

We also examined more general gender-related differences (i.e., collapsing different age groups). These tests were exploratory, but we found some evidence for basic differences in motivation for prospective memory tasks. Specifically, females were more likely to report tasks related to education and self/personal growth goals. For *concern* domains linked to tasks, gender differences emerged in three categories. Females reported more tasks related to concerns about education. In contrast, males reported more tasks related to concerns about property/possessions and travel. It is noteworthy that gender-related differences emerged in only two of 14 possible domains for goals and three of 14 domains for concerns. Therefore, we conclude that there are more gender-related similarities than differences in the goal and concern domains motivating real prospective memory tasks. When combining our results on gender group differences in motivations linked to prospective memory tasks with Nurmi’s [7] findings on listed goals and concerns, we see two consistent differences in salient motivational domains for males and females. Specifically, in the domains of education and self, females more commonly reported both goals and prospective memory tasks related to these types of goals. However, neither our results nor Nurmi’s results matched the patterns predicted by social roles for the two genders (e.g., [45, 51, 52]). For instance, in the current study, females’ tasks were *not* driven more by caregiving goals and concerns (e.g., children, marriage) and males’ tasks were *not* driven more by profession goals and concerns. As our findings were the first we know of on gender-related differences in motivation for prospective memory, replication in future studies would be beneficial.

### Older men’s prospective memory tasks are less motivated by approach (goals) and more motivated by avoidance (concerns)

The third research question was *Do younger and older adults show general differences in approach and avoidance motivation for their prospective memory tasks*? The third hypothesis was that, compared to older adults, young adults would generally have more tasks motivated by *goals*. The fourth hypothesis was speculative, but predicted that older adults would have more tasks motivated by *concerns*. Results showed that younger adults showed a marginally higher percentage of prospective memory tasks motivated by goals (approach motivation) than older adults did, but no difference in percentage of prospective memory tasks motivated by concerns (avoidance motivation).

However, as our second research question concerned gender-related differences, we tested these also. There was a significant gender by age group interaction for both goal motivation and concern motivation. Specifically, older males differed from the other three subgroups. That is, they showed nominal differences from all three other subgroups. The motivation they reported for everyday prospective memory tasks showed a pattern of relatively low approach motivation (statistically lower than all three other subgroups) and relatively high avoidance motivation (statistically higher than older females, and nominally higher than younger males and females). We interpret this set of results as meaning that the everyday prospective memory tasks of older males are actually less driven by their approach aspirations and more driven by their fears. An alternative explanation is that the different pattern observed for older males just reflects reporting differences. However, we might expect the opposite pattern if older males were responding in a way that they thought reflected their traditional role—i.e., the traditional masculine role might be seen as one in which a man does *not* say that his everyday tasks are motivated by fears.

How do these findings fit with past results? More generally, the developmental goal literature has found that, compared to younger adults, older adults appear to be less motivated by “gains” (approach motivation; (e.g., [11, 14, 60]). For example, goals such as “increasing knowledge about a topic” decrease with age. Our results extend this claim to now apply to motivation specifically driving prospective memory tasks, but we found that reduced approach motivation only occurred in older men. Previous research has shown mixed results on whether age brings increased motivation to avoid bad outcomes, that is, greater avoidance motivation (e.g., [7, 37]). We found no general age-related increase in the likelihood of prospective memory tasks being motivated by concerns, although we did find this for older males, when compared to older females. Finally, we could not find any past results on gender by age interactions in approach and avoidance motivation. Therefore, the currently found interactions make a new contribution to the literature.

The discovery that older adult males appear to be less motivated by goals and more motivated by concerns has practical implications. Other findings suggest that framing what one is trying to do in avoidance terms has deleterious effects on motivation [8, 10], performance [10], and declarative memory [58]. Older males, therefore, might be especially vulnerable to impediments in goal-directed action. Interventions designed to emphasize approach motives for remembering tasks might be especially effective with this segment of the population.

### Limitations

A limitation in the current study was the use of a cross-sectional design to examine age-related differences. Because of possible cohort effects, longitudinal research on these issues would be informative. However, even if these age-related differences in prospective memory task motivations are unique to these age groups at this point in history, these findings still provide information about the real prospective memory tasks of younger and older adults today and are therefore useful to researchers interested in prospective memory and aging. A second limitation was the inclusion of only two age groups. For example, additional research that included a middle-aged group would provide important information about prospective memory motivation in mid-life. A third potential limitation concerns the sample, which may not be fully representative of the larger populations. Specifically, all participants lived in Wyoming, a very sparsely populated, rural state in the U.S. with low rates of ethnic minorities [68], so broad generalization would require replication in samples that represented urban populations and populations with higher rates of ethnic minorities. Further, the older adults sampled in this study were living independently and constituted a fairly healthy subset of adults in that age range. Therefore, their everyday prospective memory tasks and related goals and concerns might differ from older adults who are in poor health and/or not living independently (due to financial or other limitations). Our sample was also on the small side (N = 89). Future studies that include a larger sample would allow testing of interaction effects of age and gender on motivation within categories of prospective memory tasks. Another limitation is that we did not also assess prospective memory performance. Future studies might do this by using a diary method to track planned intentions and actual performance. A final limitation was our method for operationally defining approach motivation as motivation by goals and avoidance motivation as motivation by concerns and fears. This represents just one way to measure these constructs, and so future research that uses different operational definitions would be helpful.

### Conclusion

Research on prospective memory (remembering intentions) and aging has been largely conducted by cognitive psychologists who, not surprisingly, have focused on “cold,” strictly cognitive, processes. With some exceptions (e.g., [24, 25, 27]), researchers have neglected investigating the potential “hot” influences of motivational variables. Our study suggests that motivation for prospective memory, including task choice, is different for younger and older adults. These groups have different goals and concerns which motivate their everyday prospective memory tasks, and which generally match age appropriate developmental tasks. We also found interesting age by gender interactions on measures of general task motivation (approach and avoidance motivation) that appear to be novel. Specifically, older males reported that their everyday intentions were driven less by goals to be attained, and more by negative outcomes to be avoided. Altogether, the current findings may have important implications for improving our understanding of when and why younger and older adults differ in their abilities to remember to perform intentions. More generally, we suggest that age-related differences in motivational variables underlying prospective memory need to be considered by researchers in order to conduct more ecologically valid investigations of prospective memory and aging and to inform effective memory interventions.

## Supporting information

S1 File Dataset. (XLSX)
